# Chemo-Ultrasonication Rehabilitation of Thin-Film Composite Ultrapure Water Membrane for Spent Dialysate Recovery

**DOI:** 10.3390/membranes15110340

**Published:** 2025-11-14

**Authors:** Nuhu Dalhat Mu’azu, Mukarram Zubair, Mohammad Saood Manzar, Aesha H. Alamri, Ishraq H. Alhamed, Asaad Al Alawi, Muhammad Nawaz

**Affiliations:** 1 Department of Environmental Engineering, College of Engineering, Imam Abdulrahman Bin Faisal University, Dammam 31451, Saudi Arabia; mzzubair@iau.edu.sa (M.Z.);; 2 Department of Chemistry, College of Science, Imam Abdulrahman Bin Faisal University, P.O. Box 1982, Dammam 31441, Saudi Arabia2230700017@iau.edu.sa (I.H.A.); 3 Boys Section, Rowad Al-Khaleej International School, Dammam 32423, Saudi Arabia; 4 Department of Nano-Medicine Research, Institute for Research and Medical Consultations (IRMC), Imam Abdulrahman Bin Faisal University, Dammam 31441, Saudi Arabia

**Keywords:** reverse osmosis (RO) membranes, ultrasonication cleaning, membrane rehabilitation, sustainable wastewater treatment, fouling mitigation, circular economy, uremic toxins, environmental sustainability, wastewater reclamation

## Abstract

The ever-increasing number of discarded end-of-life dialysate polyamide thin-film composite membranes (DEoLMs) from presents both environmental and economic challenges for health centers. Traditional thermo-chemical cleaning techniques have been deployed for the rehabilitation of DEoLMs. This study further investigated the application of chemo-ultrasonication rehabilitation of dialysate-production-related DEoLM for potential reuse in spent dialysate recovery considering salt and creatinine—a typical uremic toxin-removal from water. The DEoLM was rehabilitated using low-concentration citric acid (CA) and sodium lauryl sulfate (SLS) under ultrasonic waves (45 kHz, 30 min agitation). Considering different rehabilitation protocols, the synergistic effects of heating (HT) and the chemical agents, with and without and ultrasonic waves (SC) were evaluated through FTIR, SEM, and EDX analyses, and the performance of the rehabilitated DEoLM was assessed via water flux and permeance, and efficiencies for conductivity and creatinine rejection. The fully integrated protocol chemo-ultrasonication (HT + SC + chemical agents) yielded the highest performance, achieving 93.56% conductivity and 96.83% creatinine removal, with water flux of 113.48 L m^−2^ h^−1^ and permeances of 6.31 L m^−2^ h^−1^ bar^−1^, at markedly reduced pressures. The chemo-sonic-rehabilitated-DEoLM removed the organic–inorganic foulants beyond thermo-chemical cleaning. This suggests that the sonication waves had a great impact regarding rejuvenating the fouled DEoL dialysate membrane, offering a sustainable, cost-effective pathway for extending membrane life, and supporting sustainable water management to achieve circular economy goals within healthcare centers.

## 1. Introduction

Reverse osmosis (RO) membranes are widely used in water and wastewater treatment and their role in desalination is well known [1]. They are extensively utilized in hemodialysis water treatment systems and other medical applications to ensure the production of ultrapure water that complies with strict international health and regulatory standards [2,3]. These membranes are typically fabricated as polyamide thin-film composites (TFC), which provide high water permeability while effectively rejecting salts, organic compounds, and other dissolved contaminants through advanced RO separation mechanisms [4,5]. However, their lifespan is constrained by fouling and performance deterioration, leading to large volumes of end-of-life (EoL) membrane waste each year [2,6]. Traditionally, discarded membranes are incinerated or sent to landfills, posing environmental and economic challenges. To support circular economy initiatives, there is increasing interest in recovering and rehabilitating spent membranes for reuse across less demanding applications, rather than disposing of them [6].

A recent review, by Muazu et al. [2], on hemodialysis (HD) polymeric membranes emphasized the adoption of sustainable strategies for managing hemodialysis water, particularly through a closed-loop system for dialysate recycling and reuse. In alignment with global frameworks under the UN Sustainable Development Goals (SDGs) and several countries’ Vision, such as KSA Vision 2030, these initiatives advocate for eco-friendly approaches to foster a circular economy [4,7], underscoring the importance of repurposing spent membranes for alternative uses, such as for treating wastewater from used dialysate [8].

RO membrane fouling—caused by biofilms, colloidal particles, inorganic scale, and organic deposits—reduces water flux, increases energy requirements, and drives up operating costs. To enable the reuse of fouled EoLMs, cleaning and rehabilitation methods have been proposed by several of researchers [9]. This would support extending the membranes’ life spans and is more economically viable compared to opting for new RO membranes. Conventional chemical cleaning methods, such as acids, alkalis, surfactants, and chelates, are widely used to remove foulants. Yet are associated with some limitations, such as incomplete removal of tightly bound deposits, membrane damage from harsh chemicals, discharge of large chemical wastes amongst others. To address these challenges, ultrasonic-assisted method emerged as an advanced cleaning strategy that induce ultrasonic agitation to improve foulant removal and restore membrane performance [9].

Recent reported studies evidently highlight chemical ultrasonication—driven by acoustic cavitation—as a powerful and scalable approach to rehabilitate EoL RO membranes [9]. Li et al. [10] showed that oxalic acid combined with ultrasonication achieved de-fouling rates exceeding 91%, with optimized parameters (50 kHz, 0.64 W cm^−2^, and 10 min ultrasonication) and an acid–alkali sequence under ultrasound maximizing flux recovery. In another, related, work, Morales et al. [11] extended this concept by coupling sodium hypochlorite oxidation with ultrasonication, accelerating polyamide layer transformation and enabling aged membranes to function as UF-like modules for secondary treatment applications. In another relevant study, Cai et al. [12] demonstrated that lower ultrasonic frequencies (28–45 kHz) significantly outperform higher frequencies (100 kHz) in reducing reversible resistances, confirming that frequency selection is critical to maximize cavitation and acoustic streaming. Muthukumaran et al. [13] employed RSM technique to optimized operational parameters for ultrasonic cleaning protocols for dairy-fouled ultrafiltration membranes, showing that cleaning efficiency improves with careful the tuning of pH, surfactant concentration, transmembrane pressure (TMP), and ultrasonic power. In similar vein, Hoo et al. [14] emphasized the green intensification role of acoustic cavitation, demonstrating that optimizing ultrasonic parameters can dramatically enhance mass transfer, reduce reliance on harmful chemicals, and accelerate reactions through microjets, shock waves, and turbulence. Thombre et al. [15] further confirmed that low-frequency ultrasound (24 kHz, 135 W, 4 min), in combination with NaOH, achieved flux recovery of up to 90%, outperforming backflushing and chemical cleaning alone, and cautioned that excessive power or higher frequencies may damage membranes. Using hollow fiber ultrafiltration membranes, Mohamad Idris et al. [16] systematically examined the effects of ultrasonication, and reported that 15 min of ultrasound alone achieved ~57% flux recovery, while combining 1 M NaOH with 10 min ultrasonication boosted recovery to ~67%. They confirmed that acoustic cavitation, vibration, and micro-streaming loosen sludge floc structures, reduce particle size, and increase cleaning efficacy without damaging the membrane structure. Their SEM analysis revealed that sonication opened blocked pores more effectively than chemical cleaning alone. This strongly validates the use of sono-chemical approaches—simultaneous ultrasound and chemical cleaning—for synergistic foulant removal on both surfaces and pores.

Relevant reported works in this area have highlighted that ultrasonic-assisted membrane cleaning is no longer a niche technology but a rapidly advancing field, with clear evidence of scalability and broad applicability. Aghapour Aktij et al.’s [9] systematic review showed that ultrasonication effectively enhances fouling removal and permeate flux recovery across RO, UF, NF, and MF membranes by exploiting cavitation phenomena, acoustic streaming, microjets, and shock waves—physical effects that overcome foulant–membrane interaction. This vital review in the subject area stressed that integrity must be carefully managed; while ultrasound reduces chemical consumption and cleaning time, excessive power intensities can damage membranes. It recommends combining ultrasound with appropriate chemical agents (acids, alkalis, oxidizers, chelants, surfactants) to achieve synergistic effects without exceeding mechanical limits. Collectively, these studies demonstrate that chemo-ultrasonic cleaning can be customized with different chemical agents—acids like oxalic or citric, alkalis like NaOH, chelants like EDTA, surfactants like SDS, or oxidizers like NaOCl—while also optimizing ultrasonic parameters (frequency, power, and duration) to maximize fouling removal, reduce cleaning time, and extend membrane life. This adaptability enables operators to rehabilitate discarded RO membranes for a wide range of water and wastewater treatment applications, including municipal effluent polishing, industrial process water pretreatment, and brackish water filtration. Despite these advancements, there is still a lack of research exploring the specific combination of citric acid (CA), SLS, and ultrasonication for EoL RO membrane recycling. CA has been widely recognized for its ability to dissolve inorganic scaling, particularly calcium-based deposits, and is considered an environmentally friendly option due to its biodegradability while SLS, a commonly used surfactant, has shown effectiveness in breaking down organic fouling and disrupting microbial biofilms [17]. However, the efficiency of these cleaning agents are enhanced when combined with other methods, especially for heavily fouled membranes [9]. Furthermore, few studies directly compare cleaning performances across a range of metrics—including salt rejection, organic contaminant removal, and permeance recovery.

Hemodialysis centers routinely replace large quantities of EoL polyamide thin-film composite RO membranes, used in producing ultrapure dialysate water [2]. These discarded membranes represent a high-value recovery stream with strong potential for reuse if fouling can be effectively reversed. A pioneering thermo-chemical cleaning approach for rehabilitating dialysate-production-DEoLMs has recently been reported, where a wide range of chemical agents that included CA in different combinations with SDS, SLS, and ETDA were employed [18]. They reported that, a mixed CA: SLS (1:1) cleaning solution provided the most effective balance of flux recovery with high salt rejection, and creatinine removal, at reduced transmembrane pressure. Motivated by these recent advances, the present study further explores chemo-ultrasonication treatment using CA and SLS, assisted by ultrasonic agitation, to rehabilitate the highly fouled DEoLM for its second-life application in spent dialysate recovery, targeting both salt rejection and creatinine—a typical uremic toxin- from water. The physical–chemical characteristics of the fouled DEoLM determined using FTIR, SEM/EDX, and DLS were employed for elucidating the mechanism of creatinine rejection.

## 2. Materials and Methods

### 2.1. Chemical Reagents

The chemical reagent used in this study included citric acid (CA, analytical grade, ≥99.5%), sodium chloride (NaCl, analytical grade, ≥99%), sodium lauryl sulfate (SLS, laboratory grade, ≥98%) and creatinine (≥99% purity, analytical grade, Sigma-Aldrich), obtained from Sigma-Aldrich (St. Louis, MO, USA) and/or Scharlau (Cedar Grove, NJ, USA) through a local distributor and used without any further purification. The single or binary cleaning solutions used were prepared by dissolving the reagents in deionized (DI) water and mixing thoroughly to give the required concentration of each.

### 2.2. DEoLM Sample

The RO membrane modules utilized in the dialysate production unit at a hospital in Al-Khobar consisted of DuPont Water Solutions (Edina, MN, USA) (previously Dow FilmTec™) thin-film composite (TFC) polyamide membranes. The average main rated operational parameters of the pristine module are salt rejection 97–99.0%, operating pressure 600 psi (41 bar), maximum operating temperature 45 °C (113 °F), and above pH 10 limited to 35 °C. More details of the pristine module and level of compounded fouling of the DEoLM used in the present study have been reported earlier [18].

### 2.3. Chemo-Ultrasonication-Assisted Cleaning of Dialysate DEoLM

**Figure 1:**
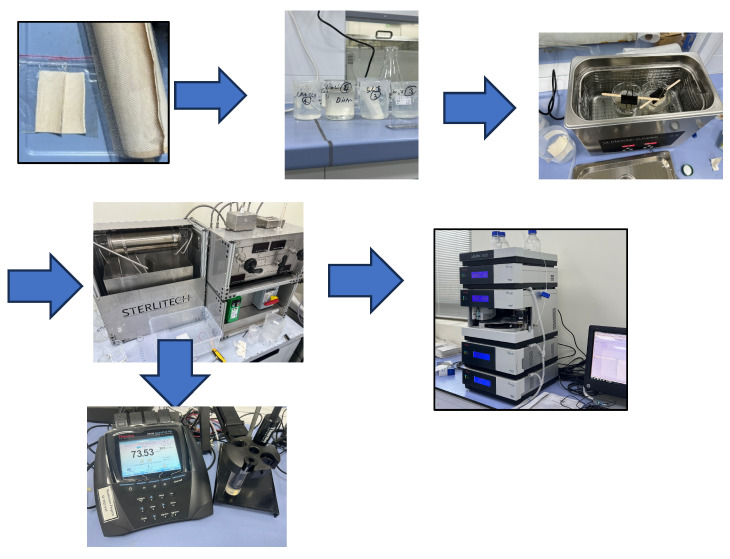
Steps of chemo-sonication rehabilitation and filtration schematic of conductivity and creatinine removal from water.

The secured DEoLM was disassembled, and the fouled thin-film composite (TFC) membrane sheets were extracted and trimmed into segments of dimensions 12 cm × 10 cm. The cut DEoLM coupons underwent chemical–ultrasonic cleaning in a digital ultrasonic bath AISI304 (Nahita, Astudillo, Spain) (120 W ultrasound power, 100 W heating power, and 45 kHz frequency) for 30 min and 45 °C fixed heating (HT) temperature with and without at sonication (SC) in combine CA and SLS solution at the following different cleaning mixture protocols: HT+CA, HT+CA+CS, HT+CA+SLS, HT+SLS+CS, HT+CA+SLS. The cleaning solution consisted of 150 mg/l (0.78 mmol) CA and 150 mg/l (0.52 mmol/L) SLS. It was ensured that the active part of the DEoLM coupons were fully immersed and firmly held in place in the cleaning solution in 200 mL glass beakers, which were placed in the ultrasonication bath to ensure uniform exposure of the ultrasonic waves. After cleaning, the membrane sheets were thoroughly rinsed with deionized water, air-dried, and then cut into smaller pieces (4.5 × 4.7 cm^2^) to fit the Sterlitech^®^ benchtop crossflow cell filtration system. The overall steps of the chemo-sonication rehabilitation and the filtration schematic of conductivity and creatinine clearance from water are illustrated in Figure 1.

### 2.4. Salt Rejection Tests

The RDEoLM obtained from the different cleaning protocols were subsequently tested for sodium chloride (1037 µS/cm average conductivity) rejection under a fixed transmembrane pressure, adjusted to maintain a constant flow rate of 0.5 gal/min. The rehabilitation effectiveness was evaluated by measuring the conductivity of the permeate, followed by detailed physicochemical characterization, as described in the subsequent section.

### 2.5. Remediation of Creatinine Contaminated Water

The performances of the different chemo-sonication RDEoLM hemodialysis membranes in the removal of creatinine were studied and evaluated using four key performance indicators, including water flux (referred to as water permeate collected from tested salt water or creatinine contaminated water), water permeance, conductivity, and creatinine removal efficiency, calculated using Equations (1)–(3).

(1) $$\text{Water flux}=\frac{V}{A\cdot t}\;L\;m^{-2}\;h^{-1}$$

(2) $$\text{Water permeance}=\frac{V}{P\cdot A\cdot t}\;L\;m^{-2}\;h^{-1}\;{\mathrm{bar}}^{-1}$$

(3) $$\text{Creatinine or conductivity removal}=\left(\frac{C_{o}-C_{t}}{C_{o}}\right)\times 100\%$$

where V = filtered permeate volume, A = membrane crossflow area, t = filtration time, P = TMP, C_o_ = initial concentration of solute, and C_t_ = final concentration of solute.

### 2.6. Creatinine Concentration and Conductivity Measurements

Creatinine concentrations in the feed water and permeate were analyzed using HPLC (Ultimate 3000, Thermo Fisher Scientific, Illkirch, France) using a Hypersil GOLD™ Amino column (Thermo Fisher Scientific, Runcorn, UK). A mobile phase of 20% deionized water and 80% acetonitrile (filtered and degassed) was run at 0.3 mL min^−1^, 30 °C, with 10 µL injections and UV detection at 220 nm. The samples were prefiltered through 0.22 µm syringe filters, and quantification was performed using calibration curves with R^2^ > 0.99. The conductivity of the feed and permeate was measured using an Orion™ Star A212 conductivity meter with a DuraProbe™ 4-pole cell (Thermo Fisher Scientific, Asheville, USA. The meter was calibrated using Orion conductivity standard solutions (84 µS/cm, 1413 µS/cm, and 12.88 mS/cm), and the readings were temperature-compensated and reported at 25 °C.

### 2.7. Physicochemical Characterization

Different techniques were employed to characterized both the DEoLM and RDEoLM samples before and after the chemical cleaning. FTIR (ATR-FTIR, Perkin Elmer, Shelton, CT, USA; 4000–500 cm^−1^) was used to assess changes in surface functional groups and chemical stability. SEM (TESCAN, Vega 3, Brno, Czech Republic) was used for the SEM-EDX analysis after gold sputter coating for examination of the surface morphology, fouling, and structural integrity and also for identification of the elemental composition and inorganic foulant distribution. Additionally, dynamic light scattering DLS (Nano ZS Malvern Zetasizer, Malvern Panalytical Ltd., Malvern, UK) analysis was performed to determine the surface charge of the membranes. These combined analyses provided a comprehensive view of the physicochemical and surface properties of the DEoLMs and RDEoLMs.

## 3. Results and Discussions

### 3.1. Characterization

#### 3.1.1. SEM Analysis

**Figure 2:**
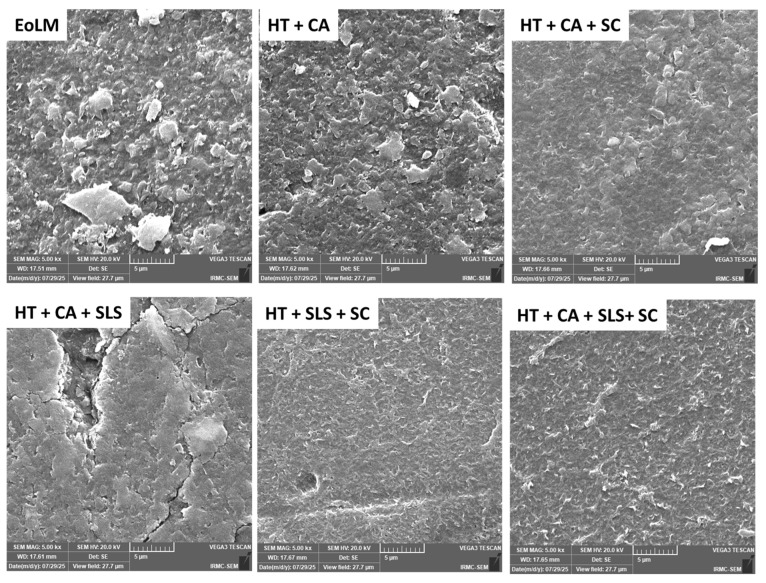
SEM images of different RDEoLM using different chemo-ultrasonic cleaning methods.

Scanning Electron Microscopy (SEM) was performed to evaluate the surface morphology of the EoL membrane before and after chemo-sonication cleaning. Figure 2 displays the microstructure of REoL RO membranes before and after the application of the various cleaning protocols. The morphology of uncleaned DEoLM showed a deposition of particles forming a thick, dense layered aggregates associated with the inorganic and organic foulants. The observed unexpectedly high degree of fouling, as reported earlier, was attributed to the damping storage environment that the DEoLM [18]. Thus, for cleaning the DEoLM using combination of HT and CA, the SEM image indicates the disappearance of some deposits which could be attributed to the surface inorganic foulant; however, the membrane surface is not fully recovered. The synergy effect of sonication in the HT+CA cleaning protocol appeared to have resulted in improvement in the membrane cleaning. This is evident in the SEM image for (HT+CA+SC) which exhibited a homogenous and more polished surface morphology with a fewer deposition of particles, which can be attributed to the accelerated mass transfer compared with HT+CA. Similarly, the SEM image of DEoLM after the cleaning protocol, HT+SLS+SC, showed a smoother surface with discrete patterns, which confirmed the effective removal of foulants from the membrane surface. Interestingly, the microstructure of the DEoLM after combined cleaning protocol HT+CA+SLS+SC indicated signs of the substantial removal of both organic and inorganic foulants, with a more clear and open membrane structure with no saturation or deposition compared to other cleaning protocols.

#### 3.1.2. EDX Analysis

**Figure 3:**
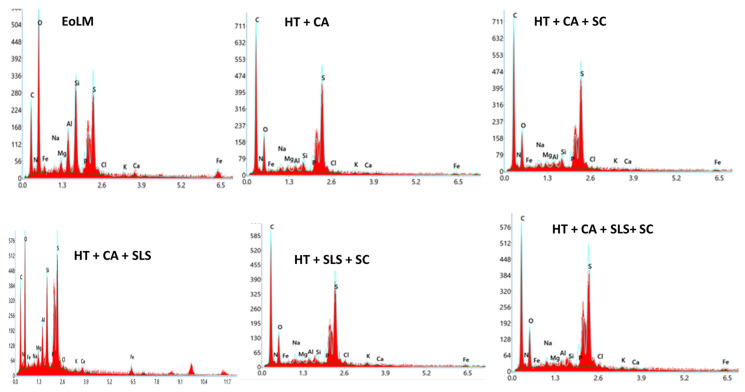
EDX Spectrums for different RDEoLM using different chemo-ultrasonic cleaning approaches.

Figure 3 and Table S1 show the variation in the elemental composition of the RDEoLM after using different cleaning protocols. As shown, the RDEoLM showed a high % weight of various elements (Si, Al, Mg, P, S, Cl, K, Ca, and Fe), mainly due to the formation of inorganic and organic foulants on the surface, as confirmed by SEM images (Figure 2). The EDX results were interpreted in a semi-quantitative manner, focusing on relative trends rather than absolute wt % values, because EDX is localized and sensitive to area selection during analysis. The presence of mixed inorganic–organic deposits was confirmed by the DEoLM’s high levels of Si, Al, Mg, P, and Fe. The relative % of these components decreased following HT+CA cleaning, suggesting a partial elimination of inorganic scale. For Mg, Al, and Si, using the HT+CA+SC cleaning protocol considerably reduced their EDX signals. Similarly, the full combined cleaning scheme (HT+CA+SLS+CS) comparitively showed lower elements compositions, illustrating its improved mineralized deposit removal from membrane surface. Moreover, the effective cleaning resulted in the exposure of the underlying polyamide layer, which is reflected in the increase in C and N. This can be further confirmed via FTIR spectra showing the attenuation of carbonate and amide-associated bands, and SEM images exhibiting a clearer membrane surface. Subsequently, there is slight increase in sulfur, which could be due to residual reductive cleaning agents and sulfur-based compounds from foulants that are still adhered inside membrane surface openings. Previous studies have reported a similar trend in polymeric membranes after cleaning and surface modifications [19,20,21].

#### 3.1.3. FTIR Analysis

**Figure 4:**
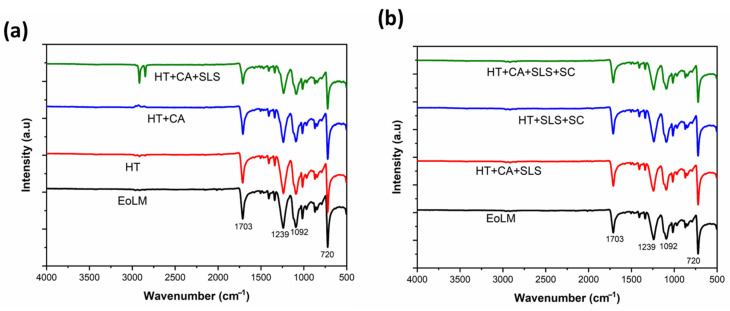
FTIR spectra of membrane under different cleaning protocols (**a**) heating and chemical, and (**b**) heating, chemical, and sonication.

Figure 4a,b displays the FTIR spectra of the RDEoLM and its rehabilitated forms under various cleaning procedures. The raw DEoLM indicated absorption bands at ~1703 cm^−1^, 1239 cm^−1^, 1088 cm^−1^, and 716 cm^−1^, which were in line with the previous studies [15]. In particular, the peaks at 1230–1080 cm^−1^ are ascribed to C–N stretching and aromatic amine contributions, whilst the band around 1700 cm^−1^ is ascribed to C=O stretching of carboxyl or amide groups [16]. The sharp band at 716 cm^−1^ is attributed to inorganic fouling deposits, such as CaCO_3_ [17]. As seen, a partial drop is observed in the intensity of the 1700 cm^−1^ band for DEoLM cleaned with HT+CA. This indicates that organic foulants (carbohydrates, proteins, and lipids) are efficiently removed by the influnece of CA chelating. Chheang et al. [22] reported comparable behavior when they used chemical agents for the cleaning of organic- and inorganic-fouled PVDF membranes. Similarly, the additon of SLS (HT+CA+SLS) promoted the reduction in the strength of peaks in the 1200–1000 cm^−1^ area, which could be attributed to the exclusion of organic foulants that are usually associated with organic/biofouling [18]. This is evident in the decrease in element O for all the cleaning protocols. Integrating sonication (HT+CA+SC and HT+CA+SLS+SC) augmented these effects, as the nucleation of tiny bubbles promoted the cleaning of extricated firmly inevitable foulant deposits. Jeong et al. [23] observed the high cleaning efficiency of a clogged filter using ultrasonic excitation, releasing shock waves which not only induced pressure but also generated high heat, thereby accelerating the cleaning performance. This was obvious from the sharper and better-resolved amide peaks, signifying the safeguarding of the polyamide active layer. The most comprehensive cleaning protol (HT+CA+SLS+SC) precisely recovered the spectrum that most diligently resembled that of the virgin polyamide RO membrane, demonstrating the effective removal of both organic and inorganic foulants while preserving the selective layer’s chemical integrity. This is also in line with the findings of SEM and EDX, presented earlier, which showed that different cleaning protocols produced lower elemental signals and the least amount of residual deposition compared with the original DEoLM [18]. Moreover, the FTIR results support earlier studies that demonstrate how chemical and ultrasonic cleaning work together to preserve polyamide functional groups and get rid of foulants in an efficient manner [20,21].

### 3.2. Performance of Chemo-Sonication-Assisted Cleaning of EoL

This section evaluates the effectiveness of chemo-sonication cleaning of DEoLM for conductivity and creatinine removal. The discussion assesses the impact of the rehabilitating strategy in synergistically removing fouling and the impact on ionic and organic rejection as well as water flux, and its permeance.

#### 3.2.1. Conductivity Removal Using Chemo-Sonication RDEoLM

**Figure 5:**
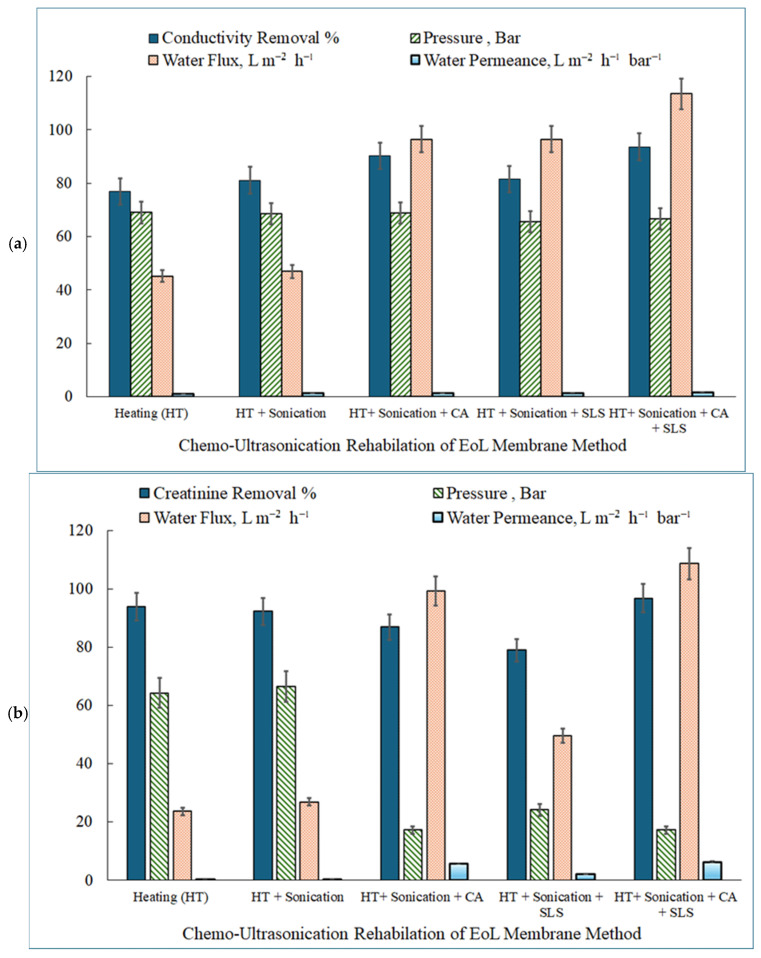
Performance of different RDEoLM using different chemo-ultrasonic cleaning (120 W; 45 kHz; 45 °C for 30 min) for (**a**) conductivity removal (1037 µS/cm; flow rate of 0.5 gal/min) and (**b**) creatinine (1.33 mmol/L; flow rate of 0.5 gal/min) removal from water.

Figure 5a illustrates the performance of RDEoLM in terms of conductivity removal, water flux, and water permeance under various investigated chemo-sonication cleaning protocols. Conductivity removal serves as a key indicator of ionic rejection efficiency, essential for applications in spent dialysate reclamation, where elevated electrolyte levels must be mitigated to produce reusable water. The conductivity removal across the chemo-ultrasonication rehabilation methods for DEoLM closely reflected the restoration of membrane filtration properties, as evidenced by the SEM, EDX, and FTIR results presented in previous section. These enhancements directly influence water flux and permeance—key indicators of hydraulic efficiency and ionic selectivity. After subjecting the DEoLM to HT only, the SEM micrographs of untreated membranes showed dense deposits of organic–inorganic complexes clogging the selective layer, indicating the partial removal of loose deposits and consistent low permeability (45.22 L m^−2^ h^−1^; 1.10 L m^−2^ h^−1^ bar^−1^), though achieving up to 76.83% conductivity removal. Introducing SC with HT increased the removal to 81.05%, as cavitation forces fragmented surface scales, promoting smoother morphologies and enhanced water pathways (flux = 46.89 L m^−2^ h^−1^; permeance = 1.24 L m^−2^ h^−1^ bar^−1^). Moreover, the presence of CA with both HT and SC significantly boosted ionic rejection (90.20%) and flux (96.45 L m^−2^ h^−1^), as CA effectively chelated divalent cations (Ca^2+^, Mg^2+^, Fe^3+^). This is consistent with Li et al. [10], who observed similar improvements in the oxalic acid + SC cleaning of polyamide RO membranes, similar to that tested in this study.

Meanwhile, when SLS replaced CA, under both HT and SC, the conductivity removal slighly declined to 81.51%, yet confirming SLS’s effectiveness as defoulant for organic scales. This observation supports the findings reported by Muthukumaran et al [13] where NaOH + SDS + SC restored 85–91% flux recovery in protein-fouled membranes. Intrestingly, the full chemo-sonic protocol (HT + Sonication + CA + SLS) in the present study achieved the highest conductivity removal (93.56%), flux (113.48 L m^−2^ h^−1^), and permeance (1.70 L m^−2^ h^−1^ bar^−1^), demonstrating the synergy between organic and inorganic foulant removal. These values surpass those reported by Thombre et al. [15] (flux ≈ 52 L m^−2^ h^−1^; rejection ≈ 99% for dyes) and Chen et al. [24] (flux = 41.1 L m^−2^ h^−1^; NaCl rejection = 96–97%), highlighting the potential for achiving superior DEoLM by combining chelation, surfactant action, and acoustic cavitation.

Similarly, the EDX analysis (Figure 3 and Table S1), indicated a decline in surface elemental fouling. This evident considering, for example, that the uncleaned DEoLM contained Mg 1.06%, Al 2.84%, Si 5.88%, Ca 0.66%, and Fe 2.60%, which predominatly decreased progressively with cleaning. Under HT+CA+SLS+US, Mg, Al, and Si were reduced to 0.46%, 0.47%, and 0.83%, respectively—consistent with the near-complete scale removal observed by Kim et al. [25] in wastewater RO membranes treated with NaOH + EDTA. These results underscore that the chemo-sonic approach effectively eliminates both inorganic and organic foulants. Considering the FTIR spectra for the untreated membranes, it exhibited strong absorption at 1703 cm^−1^ (C=O stretching from organic residues) and 716 cm^−1^ (CaCO_3_ vibration), which diminished markedly after the chemo-sonication procesess. The restored polyamide peaks mirrored those of virgin RO membranes, confirming the preservation of the selective layer, unlike in aggressive hypochlorite cleanings, reported by Morales et al. [11], that risk polymer degradation. Generally, the these results suggest that the integration of CA, SLS, moderate HT, and SC can create an effective balance between fouling removal and membrane integrity restoration. This hybrid strategy outperforms previously reported ultrasonic cleanings in flux, permeance, and ionic rejection, validating chemo-sonication as a robust, low-cost, and sustainable route for rehabilitating DEoL dialysis RO membranes for reuse in desalination or wastewater reclamation.

#### 3.2.2. Creatinine Removal from Water Using Chemo-Sonication RDEoLM

Figure 5b depicts creatinine removal trends alongside flux, TMP, and permeance metrics across the cleaning procedures investigated, highlighting the method’s potential for toxin removal from water in relation to the required TMP. Creatinine, a representative small-molecule organic toxin (~113 Da) in dialysis effluent, was used to evaluate the RDEoLM organic rejection capabilities using chemo-sonication-assisted rehabilitation methods. The remarkable gains in creatinine removal achieved through the chemo-ultrasonication cleaning protocols for DEoLM reflect a comprehensive restoration of the membrane’s functional selectivity and surface chemistry, as confirmed by SEM, EDX, and FTIR analyses. These advancements substantially improved water flux and permeance—key to maintaining hydraulic efficiency and small-solute rejection. The full combined treatment (HT+SC+CA+SLS) produced 96.83% creatinine removal, a rise that suggests the systematic elimination of organic biofilms and inorganic scales, thereby enhancing both toxin rejection and water throughout via surface and structural restoration. As presented earlier, the SEM images (Figure 2) revealed that the untreated DEoLM was densely covered with organic–inorganic deposits that blocked pores and impeded solute transport. While HT alone yielded creatinine removal of 93.93% with 23.64 L m^−2^ h^−1^ water flux and 0.37 L m^−2^ h^−1^ bar^−1^ permeance at 64.3 bar, adding SC slightly increased the flux to 26.95 L m^−2^ h^−1^ and permeance to 0.40 L m^−2^ h^−1^ bar^−1^. However, further addition of CA (i.e., HT+SC+CA) generated an exceptional hydraulic performance—99.29 L m^−2^ h^−1^ flux and 5.76 L m^−2^ h^−1^ bar^−1^ permeance only at much lower 17.2 bar—although creatinine rejection decreased to 86.93%,. This could be attributed to inorganic scale chelation and pore reopening, thereby corroborating previous reported work using chemo-sonication [10].

Interestingly, when SLS replaced CA in the HT+SC protocol, the creatinine removal dwindled to 78.99% with an associated lower flux and permeance of 49.65 L m^−2^ h^−1^; 2.06 L m^−2^ h^−1^ bar^−1^ at 24.1 bar, respectively. This could be attributed to the SLS’s role in breaking down organic biofilms but its impact is limited on inorganic scales as compared to CA. Comparable patterns were reported by Muthukumaran et al. [13] or protein (whey) removal and by Cai et al. [12] for dextran-fouled UF membranes, where SC alone enhanced flux two-fold but in some cases achieved only partial organic removal (~95% dextran rejection). Similarly, Thombre et al. [15] achieved 99% dye rejection but moderate hydraulic recovery (≈ 52 L m^−2^ h^−1^; 2.3 L m^−2^ h^−1^ bar^−1^), underscoring the challenge of restoring both selectivity and permeability simultaneously for some cleaning protocols. In contrast, the present DEoLM chemo-sonic protocol attained a unique dual improvement: creatinine removal 96.83% (comparable to dextran and dye rejection in earlier works [12,13] while achieving far higher flux (108.75 L m^−2^ h^−1^) and permeance (6.31 L m^−2^ h^−1^ bar^−1^) at moderate pressure (17.2 bar)). This demonstrates that the synergistic combination of CA+SLS+SC not only matches the selectivity seen in dye- or dextran-based membranes but surpasses them in hydraulic recovery.

The EDX analysis showed good reduction in Mg, Al, and Si (0.46%, 0.47%, and 0.83%), paralleling flux and permeance gains, while FTIR revealed the disappearance of C=O and C–N bands typical of bio-organic fouling. The reappearance of sharp amide-I and amide-II peaks mirrored the spectral pattern of pristine polyamide, confirming the restoration of the active layer’s chemistry and selective function. While related works by [12,13] have demonstrated selective removal under moderate fluxes, the present protocol delivers both high rejection (>95%) and high flux (>100 L m^−2^ h^−1^), underscoring its higher capability to remove combined organic–inorganic foulants simultaneously. Thus, corroborating earlier CA+SC+SLS chemo-ultrasonic cleaning studies, the chemo-sonic DEoLM treatment further uniquely couples high organic rejection with superior hydraulic recovery. The synergy of creatinine removal, flux, and permeance showcases the impact of integrated chemo-ultrasonication. As fouling diminishes, flow resistance drops, enabling robust flux at lower pressures, which elevates permeance and strengthens toxin rejection.

#### 3.2.3. Comparison of Ultrasonication-Assessed RO Membrane Rehabilitation Studies

Table 1 compares the chemo-sonic cleaning performance of RDEoLM from dialysis systems with those reported for various polymeric RO, UF, and NF membranes treated by ultrasonic and chemical methods. Conventional single-step chemical cleaning (e.g., NaOCl oxidation or alkaline–acid sequences) achieved limited recovery, typically restoring fluxes of 40–80 L m^−2^ h^−1^ with permeance below 2 L m^−2^ h^−1^ bar^−1^. In contrast, coupling ultrasound with chemical reagents consistently enhanced foulant detachment through cavitation, micro-streaming, and interfacial turbulence, leading to 70–95% flux recovery and sustained rejection above 90%. Among the studies reviewed herein (Table 1), Li et al. [10] reported a water flux ≈ 55 L m^−2^ h^−1^ and NaCl rejection ≈ 91% using oxalic acid ultrasonication at 50 kHz; Muthukumaran et al. [13] achieved 85–91% recovery for proteinaceous whey fouling using NaOH + SDS + US at 50 kHz. Meanwhile, Thombre et al. [15] observed 84–90% recovery for dye-fouled NF membranes under 24 kHz sonication with NaOH. In another recent work undertaken by Khoo et al. [26], chemo-ultrasonication treatment rapidly converted the EoL RO membrane into a high-flux MF membrane through the accelerated degradation of the EoLM PA layer via 15 min KMnO_4_ (5000 mg/L) + ultrasonication. They reported a pure water permeability increment from 2.45 L·m^−2^·h^−1^·bar^−1^ (for the EoLM) to 172.6 L·m^−2^·h^−1^·bar^−1^, though with significant decline in salt rejection down to 1.52%. In comparison, using ultrasonication alone, they recorded more moderate permeability of 70.68 L·m^−2^·h^−1^·bar^−1^ and a 10.36% rejection. Additionally, they achieved a higher clearance of humic acid (87%) and BSA (68%). These results confirm the strong synergistic effect of combining oxidant and ultrasound, enabling the fast, low-cost upcycling of EoL RO membranes [26]. These benchmarks demonstrate that ultrasound enhances surface renewal and mitigates chemical diffusion limitations, but that cleaning efficiency still depends on foulant type and chemical selection.

In the present study, the chemo-sonic rehabilitation of dialysate DEoLM produced a markedly higher and more balanced performance. The CA + SLS + Heat + Ultrasonication protocol yielded 93.6% conductivity and 96.8% creatinine removal, with an average water flux of 115 L m^−2^ h^−1^ and permeance up to 6.31 L m^−2^ h^−1^ bar^−1^, surpassing the comparative ultrasonic cleaning reports shown in Table 1. Moreover, the achieved output of the optimized chemo-sonication dialysate RDEoLM outperformed the thermo-chemical system using CA: SLS (1:1) reported recently [18], which exhibited hydraulic performance restoration, with flux and permeance increasing to 59.36 L m^−2^ h^−1^ and 1.79 L m^−2^ h^−1^ bar^−1^, respectively, though with slightly lower creatinine and conductivity removal [20]. The synergy of the thermo-chemical in the presence of heating stems from (i) CA chelating inorganic scales, such as CaCO_3_ and Fe-oxides, (ii) SLS emulsifying hydrophobic and proteinaceous foulants, (iii) thermal activation accelerating reaction kinetics, and (iv) acoustic cavitation providing localized shear and pore re-opening. By comparison, CA + Heat + US and SLS + Heat + US alone yielded slightly lower rejections (87% and 90%) and fluxes (~96 and 101 L m^−2^ h^−1^), confirming that dual-agent synergy is essential for complete foulant removal. Relative to similar systems, the chemo-sonic DEoLM not only restored desalination-grade ionic rejection but also maintained high organic solute clearance (creatinine) at moderate trans-membrane pressures (17–68 bar). This indicates that the hybrid cleaning rejuvenates both surface charge and porosity. The performance exceeds the oxalic acid, NaOH, and SDS ultrasonication protocols in flux and permeance while matching their rejection stability. Hence, the CA + SLS + Heat + US approach represents an optimized, multi-mechanistic, low-energy rehabilitation route, capable of repurposing discarded dialysate membranes for secondary desalination, wastewater polishing, or spent dialysate-recovery applications—advancing circular economy and zero-waste goals.

### 3.3. Comparison Between Sonication- and Heating-Assisted Cleaning of DREoL

#### 3.3.1. Conductivity Removal by RDEoLM: Heating vs. Ultrasonication

**Figure 6:**
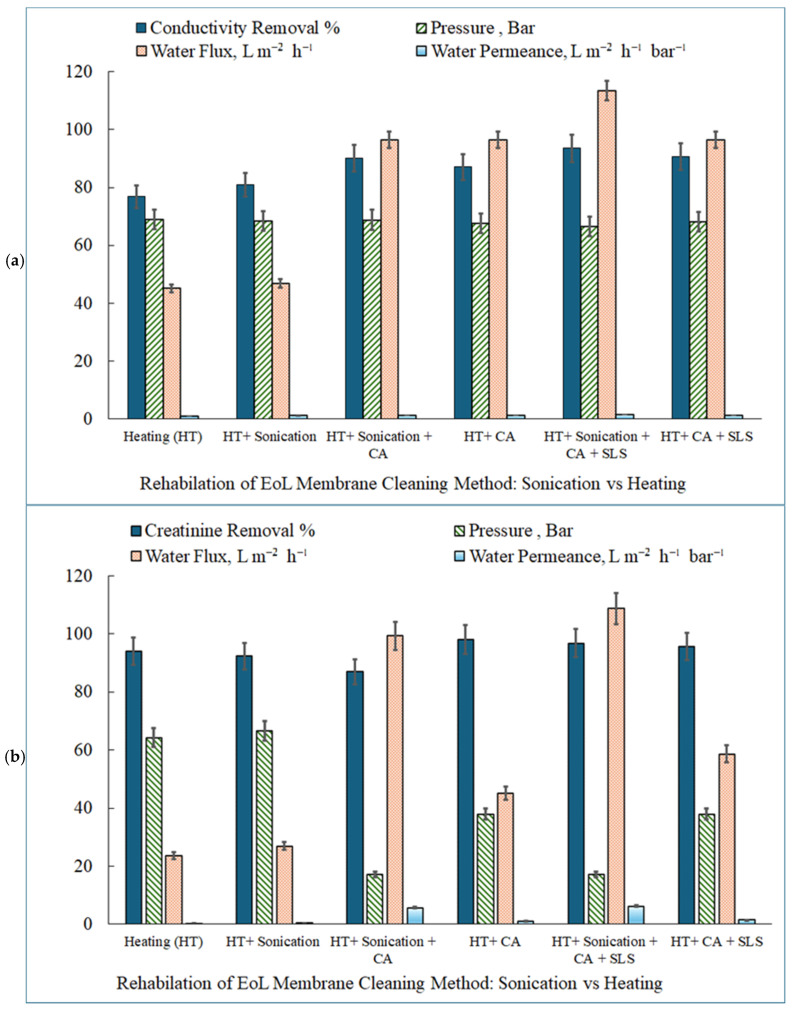
RDEoLM comparative performance of chemo-thermal (45 °C; 30 min) and chemo-ultrasonic (120 W; 45 kHz; 45 °C; 30 min) (**a**) conductivity removal (1037 µS/cm; flow rate of 0.5 gal/min) and (**b**) creatinine (1.33 mmol/L; flow rate of 0.5 gal/min) removal from water.

Figure 6a compares the performance of RDEoLM in terms of conductivity removal, water flux, and permeance under protocols involving CA and SLS, and HT with or without SC. The comparative assessment of conductivity removal by RDEoLM highlights the progressive advantages of chemo-thermal strategies and the superior efficiency of ultrasonication-assisted cleaning. HT only produced 76.83% conductivity removal, with flux = 45.22 L m^−2^ h^−1^ and permeance = 1.10 L m^−2^ h^−1^ bar^−1^. Incorporating citric acid (HT+CA) improved removal to 87.04%, doubling flux to 96.45 L m^−2^ h^−1^ and permeance to 1.42 L m^−2^ h^−1^ bar^−1^, with FTIR revealing the attenuation of the 1700 cm^−1^ carbonate band from scale dissolution. This supports An et al. [28], who found that CA applied before EDTA enhanced foulant detachment and maintained > 95% flux recovery (FRR) in TFN RO membranes under 45 °C heating. Likewise, Ochando-Pulido et al. [29] reported complete permeability restoration using a mild two-step CA + NaOH + SDS process at 30–35 °C, confirming the thermal–chemical synergy in mixed organic–inorganic fouling removal. Adding SLS (HT+CA+SLS) further increased conductivity removal to 90.69%, sustaining high flux and permeance at the 68.14 bar. Similar effects were observed by Ang et al. [30], who observed that SDS + NaOH treatments achieved > 90% flux recovery in polyamide RO membranes fouled by alginate–BSA mixtures but required high pH and prolonged contact time. Introducing ultrasonication markedly enhanced the cleaning outcomes.

The HT+SC protocol yielded 81.05% conductivity removal, flux = 46.89 L m^−2^ h^−1^, and permeance = 1.24 L m^−2^ h^−1^ bar^−1^, as SEM displayed smoother surfaces from cavitation-induced shear. When combined with CA (HT+SC+CA), removal increased to 90.20%, surpassing HT-only methods. The full chemo-sonic sequence (HT+SC+CA+SLS) achieved 93.56% removal, flux = 113.48 L m^−2^ h^−1^, and permeance = 1.70 L m^−2^ h^−1^ bar^−1^, supported by SEM’s open-pore morphology, minimal EDX residuals (Mg 0.46%), and FTIR spectra identical to virgin polyamide. The greater rejection and higher permeance compared to heating-only routes correlates with the literature showing cavitation’s role in accelerating mass transfer and chemical penetration. Garcia-Fayos et al. [31] achieved 96.8% salt removal index for SDS cleaning at 40 °C, while Gul et al. [17] observed up to 288% flux recovery using NaOH + Triton under ultrasonic agitation. These parallels affirm that acoustic micro-streaming and bubble collapse intensify reagent diffusion and foulant dislodgment, yielding superior hydraulic recovery. Overall, ultrasonication not only enhances fouling removal, by creating localized turbulence and micro-shear, but also reduces the required chemical concentration and temperature, aligning with the sustainable rehabilitation principles reported by Masse et al. [32] and Jung et al. [33]. Therefore, the present DEoLM chemo-sonic strategy demonstrates an energy-efficient improvement over conventional heat-based cleaning, achieving the highest combined conductivity removal, flux, and permeance among the reviewed protocols. Additionally, the DLS analysis employed to record the surface charge of the DEoLM recorded 4.97 ± 3.48 mV beforehand, while after the chemo-sonication cleaning the analysis showed 2.05 ± 5.32 mV. This indicates the effective removal of the charged foulants and the partial restoration of the membrane’s native surface chemistry, contributing to its improved permeability and reduced fouling potential. Thus, this further confirms that the method successfully transforms a fouled, charged EoL membrane into a clean, low-fouling surface ideal for closed-loop reuse in dialysis wastewater treatment, which supports circular economy goals in healthcare water systems.

#### 3.3.2. Removal of Creatinine via DEoLM Filtration: Heating vs. Ultrasonication

Figure 6b presents the comparative performance of RDEoLM cleaning with and without ultrasonication in relation to the achievable creatinine removal, flux, and permeance, and the TMP requirement to main the feed flow of 0.5 gal/minute. The comparative evaluation of the creatinine removal via RDEoLM reveals that while thermo-assisted chemical cleaning effectively mitigates organic fouling, its performance is notably enhanced when combined with the SC. While HT facilitates surface desorption and solute diffusion, ultrasound, on the other hand, introduces dynamic cavitation and micro-streaming that dislodge deeper foulant layers and improve the accessibility of chemical agents to the selective surface. Under heating alone (HT), creatinine removal reached 93.93%, with flux = 23.64 L m^−2^ h^−1^ and permeance = 0.37 L m^−2^ h^−1^ bar^−1^ at 64.28 bar. The addition of SLS (HT+CA+SLS) provided a stronger emulsification of organic foulants, yielding 78.99% creatinine removal, with flux = 49.65 L m^−2^ h^−1^ and permeance = 2.06 L m^−2^ h^−1^ bar^−1^ at 24.14 bar. These results comparable to those of Ang et al. [30] and Ochando-Pulido et al. [29], who observed improved organic removal when SDS or SLS surfactants were added to mild heating regimes, achieving 90–95% flux recovery and moderate rejection gains in hybrid NF/RO systems. Ultrasonication substantially amplified cleaning efficacy by generating acoustic cavitation that loosened polymeric biofilms and disrupted inorganic–organic complexes. The HT + Sonication protocol achieved 92.34% creatinine removal, with flux = 26.95 L m^−2^ h^−1^ and permeance = 0.40 L m^−2^ h^−1^ bar^−1^ at 66.55 bar, supported by SEM’s smoother surface and reduced microfouling density. When integrated with citric acid (HT+SC+CA), performance dramatically improved, achieving 86.93% removal, but flux = 99.29 L m^−2^ h^−1^ and permeance = 5.76 L m^−2^ h^−1^ bar^−1^ at 17.24 bar, illustrating the superior pore reactivation and scale dissolution. The subsequent inclusion of SLS (HT+SC+CA+SLS) delivered the highest results—96.83% creatinine removal, with flux = 108.75 L m^−2^ h^−1^ and permeance = 6.31 L m^−2^ h^−1^ bar^−1^—surpassing both the heating-only and partially sonicated methods.

These outcomes align with several ultrasonic cleaning studies targeting organic solutes. Muthukumaran et al. [13] achieved 91% flux recovery in protein-fouled polysulfone membranes using NaOH + SDS + US, while Thombre et al. [15] and Li et al. [10] observed the similar cavitation-assisted detachment of dyes and organics, though their permeances (~2.0–2.3 L m^−2^ h^−1^ bar^−1^) remained below the levels achieved here. Moreover, Garcia-Fayos et al. [31] reported 96.8% salt removal using SDS + sonication at 40 °C, paralleling the combined ionic and organic rejection observed for DEoLM chemo-sonic rehabilitation. FTIR analysis further validated these improvements. Untreated membranes displayed strong organic peaks at ~1703 cm^−1^ (C=O) and 1230–1080 cm^−1^ (C–N), which diminished under HT + Sonication + CA + SLS, restoring the characteristic amide-I and amide-II peaks indicative of an intact polyamide layer. Similar spectral regeneration after ultrasonic cleaning was noted by An et al. [28] and Masse et al. [32] in RO membranes, confirming that moderate ultrasound exposure preserves the selective layer’s chemistry while enhancing permeability. Overall, ultrasonication improved creatinine rejection by 3–5%, and permeance by nearly an order of magnitude, relative to heating-only processes. Moreover, it reduces pressures and boosts permeance. This suggest that the enhancement as result of the cavitation-induced foulant fragmentation couple with improved chemical agent penetration [18]. The observed synergy between CA chelation, SLS emulsification, and acoustic turbulence resulted in the chemo-sonic approach as superior protocol.

### 3.4. Mechanism of Removal of Creatinine Using RDEoLM

**Figure 7:**
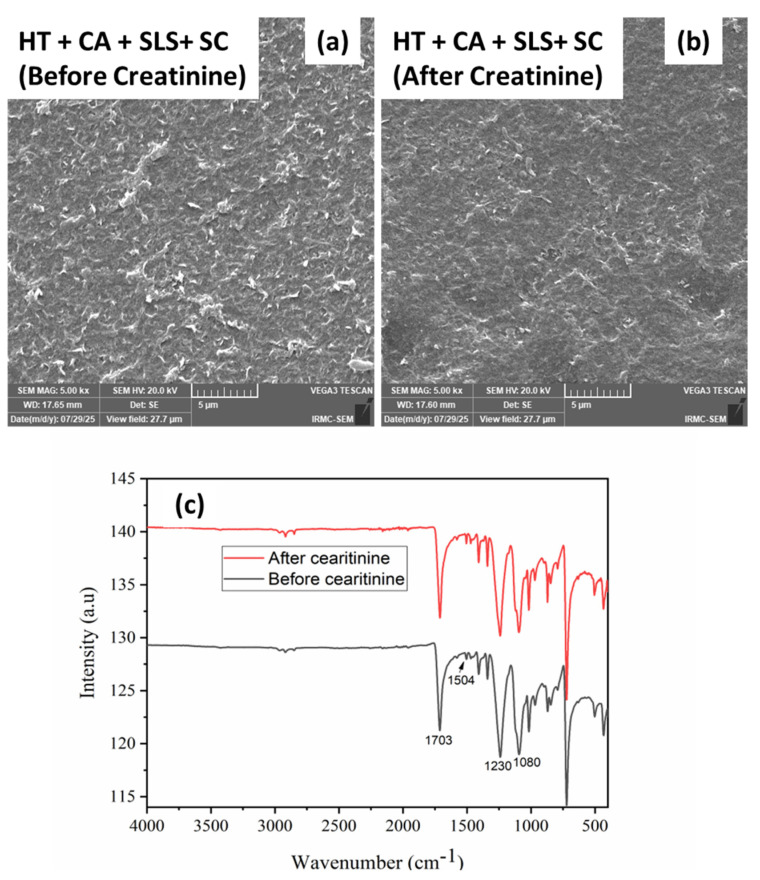
SEM images (**a**,**b**) and FTIR spectra (**c**) of cleaned DEoLM membranes using full combination before and after creatinine filtration.

The integrated morphological and chemical evidence from SEM, EDX, and FTIR analyses after creatinine filtration were invoked to elucidate the mechanism of creatinine rejection via RDEoLM. The SEM micrograph of the fully cleaned DEoLM (Figure 7a) displays a uniform, leaf-like morphology typical of an intact PA surface, confirming that the fully implemented chemo-sonic treatment (HT+CA+SLS+US) successfully removed the mixed organic–inorganic foulants, as previously reported, on aged RO membranes [28,29]. Thus, the presence of a uniform membrane structure promotes the high rejection of creatinine 96.83 (Figure 7) [34]. Similarly, EDX spectra indicated drastic reductions in Mg (0.46%), Al (0.47%), and Si (0.83%), demonstrating that scaling minerals were effectively detached by the combined chelation and cavitation effects [18,31]. This restored the compact surface function as a molecular-sieving barrier, producing the achieved 96.83% creatinine removal and flux = 108.75 L m^−2^ h^−1^ (Figure 6). After creatinine filtration, Figure 7b shows a smoother but slightly compact surface with minimal deposits, suggesting limited reversible adsorption rather than new fouling as expected [35]. The FTIR spectrum (Figure 7c) further validates this; the amide-I (≈1650 cm^−1^) and amide-II (≈ 1540 cm^−1^) bands’ characteristic peaks of polyamide, and the peaks associated with organics at ~1703 cm^−1^ (C=O) and 1230–1080 cm^−1^ (C–N), were not drastically changed to lower intensities or positions [13,15]. The foregone suggests that creatinine rejection in the rehabilitated DEoLM proceeds predominantly through steric hindrance within the re-densified PA network, assisted by electrostatic repulsion between the negatively charged surface and weakly cationic creatinine molecules [12]. The chemo-ultrasonication process re-established a defect-free, hydrophilic, and chemically stable barrier that maintains both high selectivity and permeability. Consequently, the dominant mechanism for creatinine removal in the rejuvenated EoL RO membranes is a synergistic size- and charge-exclusion process, sustained by the structurally and chemically restored polyamide layer [10,17,25]. This further corroborates the predominant mechanism of removal of creatinine via the membranes as being primarily governed by size exclusion (steric hindrance) and electrostatic repulsion within the regenerated thin-film composite (TFC) polyamide (PA) layer. This owes to the inherent small, hydrated radius (~0.34 nm) and low charge density of creatinine, which renders its separation reliance mainly on the compactness and surface charge of the PA barrier rather than on charge screening alone.

### 3.5. Performance Comparison of Chemo-Sonicated and Pristine RO Membranes

The performances of chemo-thermal reported elsewhere [18] when compared with the thermal chemo-sonication rehabilitation in the present study have distinct profiles. While chemo-thermal cleaning provides effective baseline restoration, chemo-sonication offers a more powerful, multi-mechanistic rejuvenation pathway, enabling near-complete hydraulic and selective performance recovery suitable for reuse in spent dialysate treatment and supporting circular economy membrane management. Additionally, for different categories of pristine RO-based membranes—such as Toray polyamide thin-film composites (PA-TFC), cellulose triacetate (CTA) membranes, and adsorption-enhanced mixed-matrix membranes (MMMAs)—high creatinine removal efficiencies have been consistently reported in the literature [36,37,38,39]. As reviewed by Muazu et al. [2], these materials exhibit excellent performance through optimized hydrophilicity, surface charge, and pore structure regulation, which collectively enhance water permeability and target toxins rejections. Dual-layer systems, in particular, such as CTA-based MMMAs and asymmetric hollow fiber membranes combine size exclusion and adsorptive capture, providing both physical sieving and sorbent–toxin interaction. MMMAs containing silica, zeolite, or activated-carbon fillers generally show 80–90% creatinine removal, with high permeate fluxes attributed to improved surface wettability and supplementary adsorption sites introduced by the fillers. Moreover, biopolymer membranes, such MMMAs fabricated from biomass and chitosan/starch, offer sustainable alternatives, albeit with abridged adsorption capacities [36,39]. When compared to these advanced pristine membranes, the chemo-ultrasonically rehabilitated EoL RO membranes in this study achieved comparable performance for creatinine removal from water.

## 4. Conclusions

The chemo-ultrasonication rehabilitation protocols investigated in this present study demonstrated effectiveness in restoring the hydraulic and separation performance of DEoLM compared with individual heating (HT), chemical, or ultrasonic (SC) cleaning alone. The integrated protocol (HT+CA+SLS+SC) achieved up to 96.83% creatinine removal and 93.56% ionic (conductivity) rejection, while restoring 80–90% of the hydraulic performance of virgin polyamide thin-film composite membranes. The recovered membranes exhibited fluxes up to 113.48 L m^−2^ h^−1^ and permeance values of 6.31 L m^−2^ h^−1^ bar^−1^, operating at significantly reduced transmembrane pressures (~17.2 bar). FTIR, SEM, and EDX characterizations confirmed the removal of organic and inorganic foulants and the preservation of the polyamide active layer, indicating that the chemo-sonic action enhanced foulant detachment beyond that of thermo-chemical cleaning alone. Additionally, DLS analysis revealed the surface charge of the membranes changing from +4.97 to +2.05 mV, which also indicates effective removal of charged foulants and the partial restoration of the membrane’s native surface chemistry, contributing to its improved permeability and reduced fouling potential. The dominant mechanism for creatinine removal in the rejuvenated EoL RO membranes is a synergistic size- and charge-exclusion process, sustained by the structurally and chemically restored polyamide layer. By achieving high solute rejection and hydraulic recovery with a lower energy demand, this approach offers a practical circular economy pathway to extend membrane lifespan, reduce dialysis-related solid waste generation, and lower operational costs for dialysate water treatment systems. These findings support the feasibility of repurposing EoL dialysate RO membranes for secondary treatment and spent dialysate recovery applications. Future research should be focused on examining long-term performance stability, cleaning cycle repeatability, and techno-economic analysis to support the scale-up toward full-scale healthcare and wastewater reuse operations.

## Figures and Tables

**Table 1:** Comparative chemo-ultrasonication membranes cleaning studies.

#	Membrane Type/Material	Fouling Type/Feed	Cleaning Method	Cleaning Agents	Rejection (Targeted Compound/Solute, %)	Performance (Flux/Permeance + Recovery)	Reference
1	Polyamide RO (TM720-370 and BW30-400, EoL)	Mixed inorganic/organic (Fe, CaCO_3_, silicate + organics)	Oxalic acid + Ultrasonication50 kHz, 10 min @ 43 °C (after 60 min water)	0.2% Oxalic acid (pH 2.5)	Fe^3+^ / CaCO_3_ / silicate organics 91%	Flux: 55 L m^−2^ h^−1^, Permeance: 2.1 L m^−2^ h^−1^ bar^−1^	[10]
2	Hydranautics SWC6-LD polyamide TFC (EoL RO)	Mixed organic + inorganic (seawater)	NaOCl oxidation	5% NaOCl	NaCl pre 47%, NaCl post 10%	Flux: 40–80 L m^−2^ h^−1^, Permeance: 6.9 L m^−2^ h^−1^ bar^−1^	[11]
3	Hydranautics SWC6-LD polyamide TFC (EoL RO)	Mixed organic + inorganic (seawater)	NaOCl + Ultrasonication40 kHz, 208 × 10^3^ ppm·h intensity	5% NaOCl	NaCl pre 17%, NaCl post 5%	Flux: 50–115 L m^−2^ h^−1^, Permeance: 9.05 L m^−2^ h^−1^ bar^−1^	[11]
4	Polyethersulfone (PES) UF (30 kDa)	Dextran gel layer	28–100 kHz, 20 min @ 0.8 bar	DI water	Dextran > 95%	Flux: L m^−2^ h^−1^ | Permeance: ≈4.5 @ 28 kHz L m^−2^ h^−1^ bar^−1^	[12]
5	Polysulfone UF (30 kDa, whey fouled)	Proteinaceous (dairy fouling)	NaOH + SDS + Ultrasonic 50 kHz, 10 min @ 25–55 °C, 55 kPa	0.1 M NaOH + 15 mM SDS		Flux: nan (91% recovery) L m^−2^ h^−1^ | Permeance: 4.8 @ 55 °C L m^−2^ h^−1^ bar^−1^	[13]
6	Hollow fiber UF (EoL activated sludge)	Biological + colloidal cake	Ultrasonic 28 kHz, 60 W, 10–15 min @ 30 kPa TMP ± NaOH	1 M NaOH/DI water		Flux: nan (57% recovery) L m^−2^ h^−1^ | Permeance: 3.8–4.5 L m^−2^ h^−1^ bar^−1^	[16]
7	Polyamide NF (flat sheet, 14.6 cm^2^)	Dyes (DB155, Blue150) + petroleum effluent	Ultrasonic cleaning24 kHz, 135 W, 3 min	DI water	Dyes (DB155, Blue150) 99%	Flux: 47 L m^−2^ h^−1^, Permeance: 2 L m^−2^ h^−1^ bar^−1^	[15]
8	Polyamide NF	Synthetic petroleum effluent	24 kHz, 135 W, 4 min	1 M NaOH + US	Dyes (DB155, Blue150) 99%	Flux: 52 L m^−2^ h^−1^, Permeance: 2.3 L m^−2^ h^−1^ bar^−1^	[15]
9	Vontron HP18122-50 (RO, FGD WW)	Mixed inorganics (SO_4_^2−^, Cl^−^, Ca^2+^, Mg^2+^, NH_4_^+^)	Ultrasonic cleaning (physical) 25 kHz, 3.1 W cm^−2^, 10 min @ 0.5 MPa	-	NaCl pre 94–95%, NaCl post 96–97%	Flux: 34.6–41.1 L m^−2^ h^−1^, Permeance: 2.3 L m^−2^ h^−1^ bar^−1^	[24]
10	End-of-Life RO (TFC-PA/PSf)	Brackish water fouling	Ultrasonication (15 min, 40 kHz)	5000 mg/L KmnO_4_	NaCl rejection ≈ 1.52%	Water flux 70.68 L·m^−2^·h^−1^·bar^−1^ Permeance 8–12 L/m 2.h.bar	[26]
11	Toray TM820E (EoL WW reclamation)	Organic + biofilm + colloidal	Chemical cleaning (30–40 °C, 30 min each stage)	NaOH + citric acid + EDTA	Organic + colloidal solutes 98%	Flux: 45 L m^−2^ h^−1^, Permeance: 1.62 L m^−2^ h^−1^ bar^−1^	[27]
12	TFC dialysate RO membrane	Mixed organic + inorganic + proteinaceous	CA + SLS + Heat + Ultrasonication 45 kHz, 30 min, 45 °C, 120 W	1:1 Citric acid + SLS (0.1 M each)	Conductivity 93.56%, Creatinine 96.83%	Flux: 113.48 / 108.75 L m^−2^ h^−1^, Permeance: 1.70 / 6.31 L m^−2^ h^−1^ bar^−1^	Present study
13	TFC dialysate RO membrane	Mixed organic + inorganic + proteinaceous	CA + Heat + Ultrasonication 45 kHz, 30 min, 45 °C, 120 W	Citric acid (0.1 M)	Conductivity 87.04%	Flux: 96.45 L m^−2^ h^−1^, Permeance: 1.42 L m^−2^ h^−1^ bar^−1^	Present study
14	TFC dialysate RO membrane	Mixed organic + inorganic + proteinaceous	SLS + Heat + Ultrasonication 45 kHz, 30 min, 45 °C, 120 W	SLS (0.1 M)	Conductivity 90.69%	Flux: 101.23 L m^−2^ h^−1^, Permeance: 1.52 L m^−2^ h^−1^ bar^−1^	Present study

## Data Availability

The original contributions presented in this study are included in the article and Supplementary Materials. Further inquiries can be directed to the corresponding author.

## Supplementary Materials

The following supporting information can be downloaded at: https://www.mdpi.com/article/10.3390/membranes15110340/s1, Table S1: Elemental composition by weight for rehabilitated membrane before and after cleaning.
