# Supplementary material for: Chemo-Ultrasonication Rehabilitation of Thin-Film Composite Ultrapure Water Membrane for Spent Dialysate Recovery
Source: Membranes (Basel). 2025 Nov 14;15(11):340. doi: 10.3390/membranes15110340 (PMC12654157; doi:10.3390/membranes15110340)
Supplement: Supplementary file 1 [file membranes-15-00340-s001.zip › membranes-3946770-supplementary.pdf]

Supplementary Material

Table S1: Elemental composition by weight for rehabilitated membrane before and after cleaning.

| **Element** | DEoLM | Cleaning method | | | | |
|---|---|---|---|---|---|---|
| | | **HT+CA** | **HT+CA+SC** | **HT+CA+SLS** | **HT+SLS+SC** | **HT+CA+SLS+SC** |
| C | 30.59 | 38.74 | 62.18 | 35.36 | 61.85 | 59.02 |
| N | 4.29 | 4.04 | 3.80 | 4.61 | 3.65 | 4.60 |
| O | 40.70 | 32.26 | 17.95 | 34.81 | 16.86 | 18.30 |
| Na | 0.89 | 1.01 | 0.92 | 0.72 | 1.37 | 1.13 |
| Mg | 1.06 | 0.88 | 0.54 | 0.98 | 0.51 | 0.46 |
| Al | 2.84 | 1.86 | 0.61 | 2.41 | 0.52 | 0.47 |
| Si | 5.88 | 3.65 | 0.88 | 5.06 | 0.68 | 0.83 |
| P | 1.60 | 2.15 | 1.45 | 2.09 | 1.37 | 1.86 |
| S | 8.09 | 11.74 | 9.75 | 10.38 | 10.31 | 10.92 |
| Cl | 0.28 | 0.49 | 0.28 | 0.41 | 0.58 | 0.49 |
| K | 0.51 | 0.58 | 0.43 | 0.35 | 0.69 | 0.42 |
| Ca | 0.66 | 0.65 | 0.27 | 0.63 | 0.35 | 0.33 |
| Fe | 2.60 | 1.95 | 0.94 | 2.18 | 1.26 | 1.16 |
